# Survey by the French Medicine Agency (ANSM) of the imaging protocol, detection rate, and safety of ^68^Ga-PSMA-11 PET/CT in the biochemical recurrence of prostate cancer in case of negative or equivocal ^18^F-fluorocholine PET/CT: 1084 examinations

**DOI:** 10.1007/s00259-020-05086-1

**Published:** 2021-01-08

**Authors:** Yanna-Marina Chevalme, Lotfi Boudali, Mathieu Gauthé, Caroline Rousseau, Andrea Skanjeti, Charles Merlin, Philippe Robin, Anne-Laure Giraudet, Marc Janier, Jean-Noël Talbot

**Affiliations:** 1 Direction des médicaments en oncologie, hématologie, transplantation, néphrologie, thérapie cellulaire, produits sanguins, et radiopharmaceutiques, Agence Nationale de Sécurité du Médicament et des produits de santé (ANSM), 143 Bd Anatole, F93200 St Denis, France; 2 Service de médecine nucléaire, Hôpital Tenon, AP-HP Sorbonne Université, Paris, France; 3 Nuclear Medicine Unit, ICO René Gauducheau, CNRS, Inserm, CRCINA, Nantes University, F-44000 Nantes, France; 4 Nuclear Medicine Department, Hospices Civils de Lyon, EA 3738, Université Claude Bernard Lyon 1, Lyon, France; 5 Nuclear Medicine Department, Cancer Center Jean PERRIN, Clermont-Ferrand, France; 6 Service de Médecine Nucléaire, EA 3878 (GETBO), Centre Hospitalier Régional et Universitaire de Brest, Université de Bretagne Occidentale, Brest, France; 7 Nuclear Medicine Department LUMEN, Léon Bérard Cancer Center, Lyon, France; 8 Comité permanent de l’ANSM, Médicaments de diagnostic et de médecine nucléaire, St Denis, France

**Keywords:** 18F-Fluorocholine (FCH), 68Ga-PSMA-11, Biochemical recurrence of prostate cancer, Detection rate, Evaluation of diagnostic strategy, PSA serum level

## Abstract

**Introduction:**

Despite growing evidence of a superior diagnostic performance of ^68^Ga-PSMA-11 over ^18^F-fluorocholine (FCH) PET/CT, the number of PET/CT centres able to label on site with gallium-68 is still currently limited. Therefore, patients with biochemical recurrence (BCR) of prostate cancer frequently undergo FCH as the 1st-line PET/CT. Actually, the positivity rate (PR) of a second-line PSMA-11 PET/CT in case of negative FCH PET/CT has only been reported in few short series, in a total of 185 patients. Our aims were to check (1) whether the excellent PR reported with PSMA-11 is also obtained in BCR patients whose recent FCH PET/CT was negative or equivocal; (2) in which biochemical and clinical context a high PSMA-11 PET/CT PR may be expected in those patients, in particular revealing an oligometastatic pattern; (3) whether among the various imaging protocols for PSMA-11 PET/CT used in France, one yields a significantly highest PR; (4) the tolerance of PSMA-11.

**Patients and methods:**

Six centres performed ^68^Ga-PSMA-11 PET/CTs during the first 3 years of its use in France. Prior to each PET/CT, the patient’s data were submitted prospectively for authorisation to ANSM, the French Medicine Agency. The on-site readings of 1084 PSMA-11 PET/CTs in BCR patients whose recent FCH PET/CTs resulted negative or equivocal were pooled and analysed.

**Results:**

(1) The overall PR was 68%; for a median serum PSA level (sPSA) of 1.7 ng/mL, an oligometastatic pattern (1–3 foci) was observed in 31% of the cases overall; (2) PR was significantly related to sPSA (from 41% if < 0.2 ng/mL to 81% if ≥ 2 ng/mL), to patients’ age, to initial therapy (64% if prostatectomy vs. 85% without prostatectomy due to frequent foci in the prostate fossa), to whether FCH PET/CT was negative or equivocal (PR = 62% vs. 82%), and to previous BCR (PR = 63% for 1st BCR vs. 72% in case of previous BCR); (3) no significant difference in PR was found according to the imaging protocol: injected activity, administration of a contrast agent and/or of furosemide, dose length product, one single or multiple time points of image acquisition; (4) no adverse event was reported after PSMA-11 injection, even associated with a contrast agent and/or furosemide.

**Conclusion:**

Compared with the performance of PSMA-11 PET/CT in BCR reported independently of FCH PET/CT in 6 large published series (*n* > 200), the selection based on FCH PET/CT resulted in no difference of PSMA-11 PR for sPSA < 1 ng/mL but in a slightly lower PR for sPSA ≥ 1 ng/mL, probably because FCH performs rather well at this sPSA and very occult BCR was over-represented in our cohort. An oligometastatic pattern paving the way to targeted therapy was observed in one fourth to one third of the cases, according to the clinico-biochemical context of the BCR. Systematic dual or triple acquisition time points or administration of a contrast agent and/or furosemide did not bring a significant added value for PSMA-11 PET/CT positivity and should be decided on individual bases.

## Introduction

The nuclear medicine team in Heidelberg reported in 2014 that PET/CT with PSMA-11 (also known as PSMA-HBED-CC), a ^68^Ga-labelled ligand of the prostate-specific membrane antigen (PSMA), can evidence prostate cancer (PC) tissue undetected with ^18^F-fluorocholine (FCH) [1]. The excellent diagnostic performance of PSMA-11 as the first-line PET/CT to detect biochemical recurrence (BCR) of PC has now been assessed in large series by different teams [2–9]; a few head-to-head comparative studies also confirmed its superior diagnostic performance over FCH [1, 10, 11]. But PSMA-11 is labelled with gallium-68, the 68-min half-life of which actually imposes to practice on-site labelling that requires trained personnel, ^68^Ge/^68^Ga generator, and dedicated devices. The experience and materials for ^68^Ga labelling are still currently only available in a limited number of PET centres, in France, and in several other countries. The limited activity of the ^68^Ge/^68^Ga generator results in restricting the number of patients who can be imaged with the radiopharmaceutical agent obtained from one labelling session. In contrast, industrially produced ^18^F-labelled FCH can be delivered in the same area as other ^18^F-labelled agents, at a sufficient activity for administration to patients over several hours.

Therefore, information is still pertinent and a large series is pending about the actual added value of referring a BCR patient, whose FCH PET/CT has been non-conclusive, to one centre performing PSMA-11 PET/CT. To the best of our knowledge, some information on this topic can currently be derived only from a few short series [12–15] in a total of 168 BRC patients with a previous negative FCH PET/CT.

Data from large series concerning the added value of PSMA-11 PET/CT in case of non-conclusive FCH PET/CT are particularly lacking in BCR patients with a serum level of prostate-specific antigen at the time of PET/CT (sPSA) greater than 2 ng/mL. Actually FCH PET/CT has a high positivity rate (PR) in this context and the question was raised: will a lack of evocative FCH foci generally correspond to a lack of PSMA-11 foci?

Since PSMA-11 is not yet registered for clinical use in France, the National Agency for Medicine Security (ANSM) granted nominative authorisations (ATUs) on a per-patient basis. As FCH had been registered for imaging PC since 2010 in France, ATUs can only be delivered for patients whose previous FCH PET/CT yielded a negative or equivocal result. In the ATU agreement, it was specified that the applicant must return information to ANSM about the imaging protocol, tolerance and efficacy of this diagnostic agent in each patient. All information obtained during the first 3 years of issuing ATUs was gathered, checked, and analysed by ANSM.

The main objective of this study was to determine the PR of PSMA-11 PET/CT, on per-patient and per-region approaches, in this large cohort of BCR PC, taking into account that patients were referred to PSMA-11PET/CT on basis of a negative or equivocal result of FCH PET/CT. PR was compared according to the sPSA, the initial Gleason score (GS), the modality of initial radical treatment, the patient’s age, whether FCH PET/CT was negative or equivocal, and whether the current BCR was the 1st one or the patient experienced previous BCR.

Furthermore, the ATUs from ANSM authorised the administration of PSMA-11 as a diagnostic radiopharmaceutical but did not impose an imaging protocol for the practice of PSMA-11 PET/CT. The imaging protocol was in fact rather different from one centre to another. In a large cohort, this variability was actually a strength that permits the comparison of the PR between different imaging protocols, the optimisation of which is currently the object of a large debate.

## Patients, material, and methods

### Patients and inclusion criteria in the study (**Table**1)

From May 2016 until April 2019, 1349 ATUs were granted by ANSM for PSMA-11. The ATU was not followed by PSMA-11 PET/CT in 76 cases: 38 patients refused or did not show up for PET/CT; in 15 cases, the PET/CT was done in another centre or as part of a clinical trial; in 22 cases, the diagnostic strategy was modified by the referring physician; and in 1 case, the patient died before PET/CT. One hundred eighty-nine PSMA-11 PET/CTs were performed for PC but did not match the inclusion criteria for this study: 95 were performed in another context than BCR (12 at staging, 54 for restaging recurrence, and 29 for treatment follow-up); in 39 cases, the result of FCH PET/CT was not negative or equivocal but positive and discrepant with other results of the work-up, or in 55 cases, FCH PET/CT has been performed more than 100 days prior to PSMA-11 PET/CT.

**Table 1:** Patients’ characteristics

	Nb PSMA-11 PET/CTs	Mean	Median	Range
**Age at PSMA-11 PET/CT (years)**	1084	69.3	69.7	43–89
**Gleason score**	1007	7.1	7.0	4–10
**Serum PSA level for PSMA-11 PET/CT (ng/mL)**	1084	3.3	1.7	0.03–112
**Delay between the evaluation of FCH PET/CT by thereferring physician and the PSMA-11 PET/CT (days)**	1084	45	42	4–100
	**Nb PSMA-11 PET/CTs**	**Proportion**	**Date of 1st PSMA-11 PET/CTin the cohort**	
**PET centre**	1084			
Hôpital Tenon, Paris	511	47%	May 2016	
ICO René Gauducheau, St Herblain	200	19%	July 2017	
Hôpital Lyon Sud	118	11%	July 2017	
CHRU de Brest	104	10%	October 2018	
Centre Jean Perrin, Clermont-Ferrand	88	8%	July 2017	
Centre Léon Bérard, Lyon	63	6%	October 2017	
**Gleason score**	1007			
< 7	166	16%		
= 7	660	66%		
> 7	181	18%		
**Initial therapy**	1084			
Prostatectomy (PX)	877	81%		
Other curative modality	207	19%		
**History of PC recurrence**	1084			
This BCA is the 1st recurrence	433	40%		
Previous recurrence(s)	651	60%		
**FCH PET/CT**	1084			
Negative	924	85%		
Equivocal	160	15%		

The PSMA-11 PET/CTs eligible for the analysis were performed in 6 centres in a total of 1052 patients referred for BCR after a recent FCH PET/CT yielding a negative or equivocal result. Those patients underwent 1084 examinations, since PSMA-11 PET/CT was performed twice in 32 patients. Characteristics of the patients when they were referred to PSMA-11 PET/CT for BCR are reported in **Table** 1. sPSA was assayed in relation to PSMA-11 PET/CT, either on the day of the examination or previously, at the earliest when the request for ATU was sent to ANSM.

### PET/CT acquisitions

PSMA-11 was provided by Iason (Graz, Austria) to all centres; ^68^Ge/^68^Ga generators were provided by either GalliaPharm (Eckert Ziegler, Berlin, Germany) or GalliAd (IRE, Fleurus, Belgium). Images were acquired using a time-of-flight PET/CT machine manufactured by Siemens (Erlangen, Germany), by Philips (Amsterdam, The Netherlands), or by General-Electric (Boston, USA) in 55%, 26%, and 19% of the examinations, respectively. No PET/MRI was performed. The mean injected activity of ^68^Ga-PSMA-11 was 151 MBq (median 153, range 52–328); the mean ponderal activity was 1.8 MBq/kg of body mass (median 1.9, range 0.5–3.6). No CT contrast medium was used in 74% of the examinations, an intravenous contrast medium was injected in 19% of cases, an oral contrast medium was administered in 7% of cases, but both contrast media were never administered altogether. Furosemide was administered in 488 cases (45%).

The mean CT dose-length product was 624 mGy cm (median 609, range 209–2786).

**Fig. 1:**
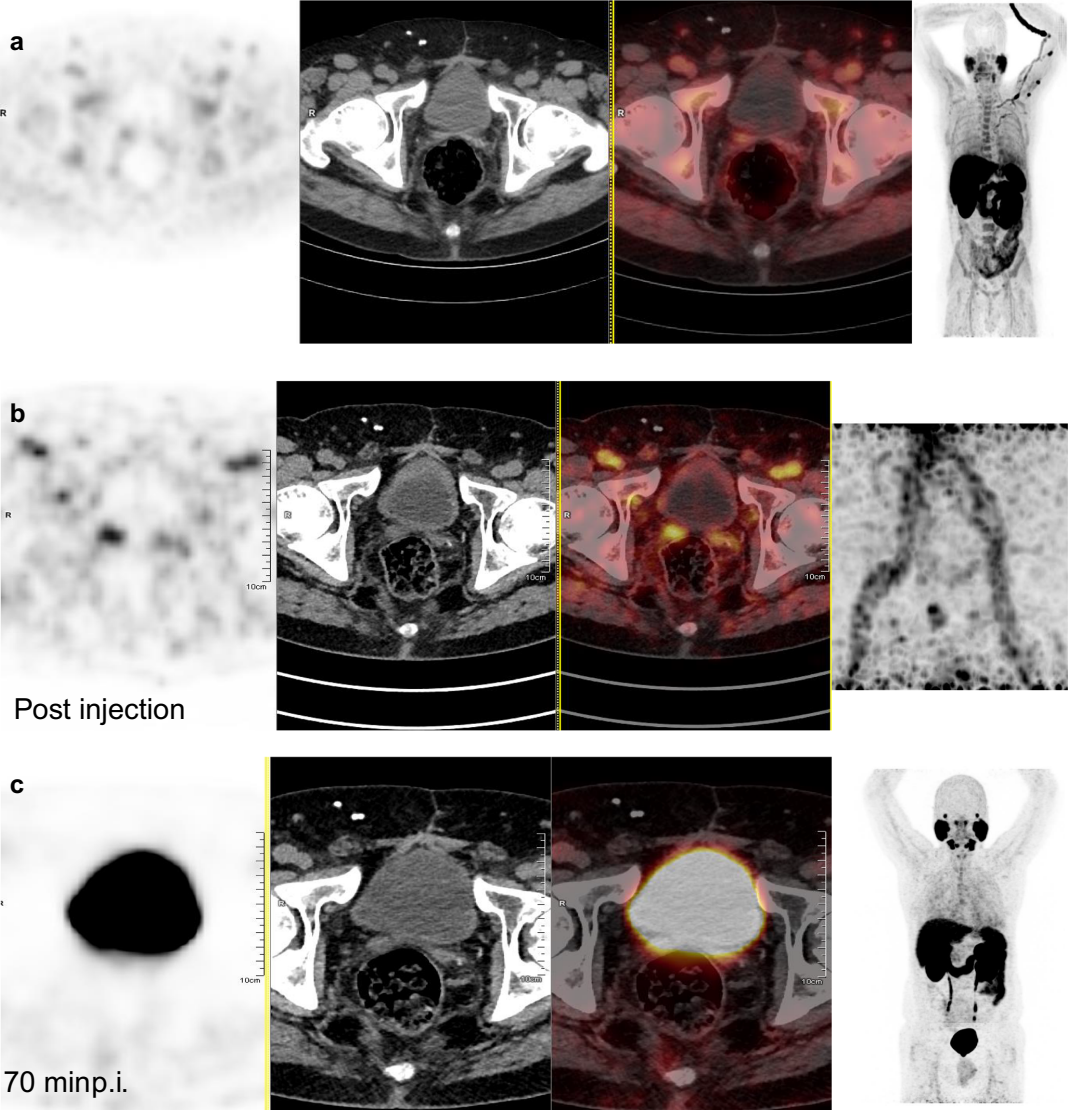
This 65-year-old patient underwent ^18^F-fluorocholine (FCH) PET/CT for biochemical recurrence of prostate cancer (BCR) sPSA = 3 ng/mL, 9 years after total prostatectomy (Gleason score 3 + 4, pT3b N0 M0). **a** From left to right: PET, CT, PET/CT fusion, MIP. FCH PET/CT showed foci of moderate intensity in the right seminal vesicle (SUVmax = 2.3), and in left ilio-obturator (SUVmax = 3.3) and right external iliac lymph nodes (SUVmax = 2.6). Overall, the result of FCH PET/CT was considered equivocal. **b** PSMA-11 PET/CT was requested before proposing a new treatment. Its early dynamic images showed a clear focus in the right seminal vesicle (SUVmax = 7.7), without any abnormal foci in the two lymph nodes that mildly took up FCH. **c** Due to the urinary excretion of PSMA-11 and the high activity in the bladder, the focus in the right seminal vesicle was no longer visible on the images acquired 70 min post-injection. Radiation therapy limited to the prostate lodge and the right seminal vesicle was completed 3 months later; sPSA was < 0.01 ng/mL and remains undetectable 2 years later. This case illustrates the added value of early acquisition for PSMA-11 PET/CT to avoid the interference of the radioactive bladder, and its specificity to rule out invasion of lymph nodes, since no recurrence was confirmed in the FCH-equivocal PSMA-11-negative pelvic lymph nodes after more than 2 years

**Fig. 2:**
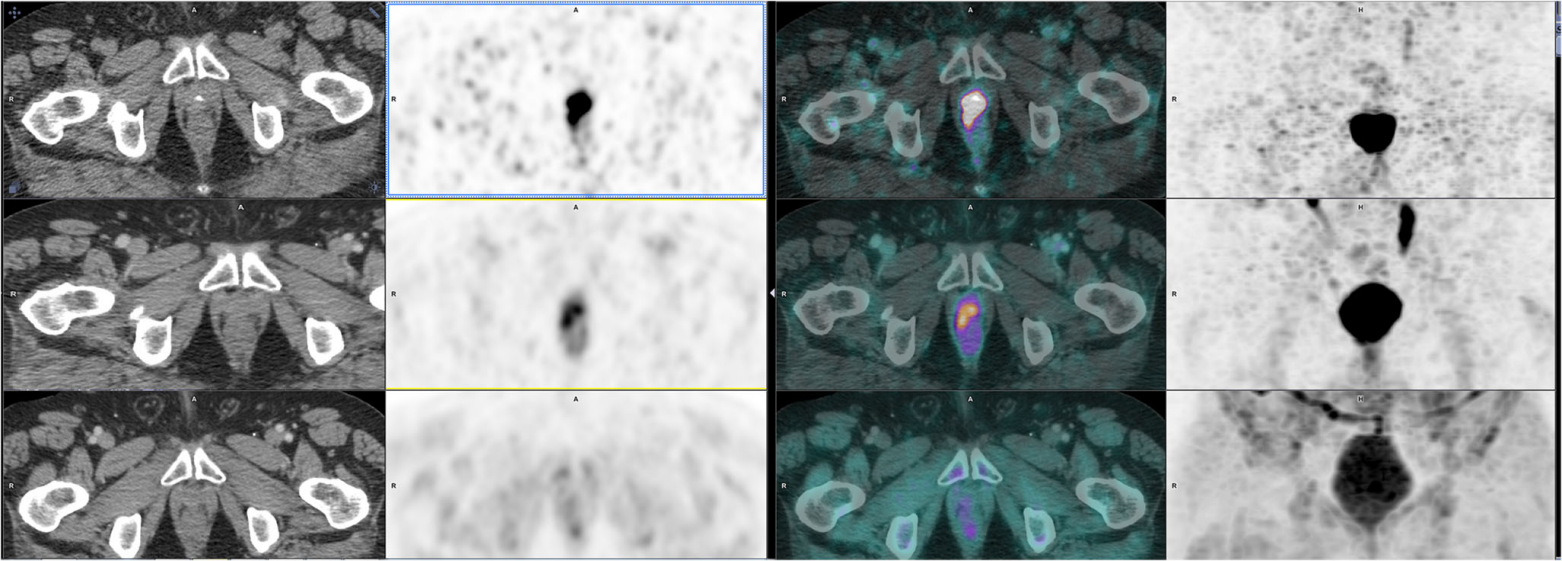
This 64-year-old patient underwent FCH PET/CT for BCR (sPSA = 0.67 ng/mL), 3 years after total prostatectomy (pT3 N0 M0). **a** FCH PET/CT (lower row of images; from left to right: CT, PET, fused PET/CT fusion, and MIP) showed a mild doubtful focus (SUVmax = 3.55) in the urethral anastomosis on the right side. **b** ^68^Ga PSMA-11 PET/CT at 59 min post-injection (middle row of images) showed an intense pathological focus (SUVmax = 6.22) at the same location. **c** The intensity of this focus increased on the delayed acquisition 158 min post-injection (SUVmax = 11.66) (upper row of images). MRI confirmed the local relapse; the patient underwent prostatic external radiation therapy with a subsequent drop in sPSA to 0.06 ng/mL after 29 months of follow-up

The number of sequence(s) of image acquisitions per PET/CT examination was as follows:

- one single PET/CT acquisition between 50 and 100 min after injection was performed in 254 cases (23%);
- dual acquisitions consisted either in a first acquisition just after injection followed by one acquisition between 50 and 100 min in 541 cases (50%) (**Fig.** 1**)** or after 100 min in 38 cases (4%), or in a first acquisition between 50 and 100 min followed by another late acquisition in 213 cases (20%) (**Fig.** 2); and
- triple time point acquisitions were performed in 38 cases (4%)*.*

Image acquisition was thus performed almost immediately after injection in 617 PET/CTs (57%), between 50 and 100 min in 1009 PET/CTs (93%), and after 100 min or as a complementary acquisition in 289 PET/CTs (27%), this late acquisition occurring on average 128 min post-injection (p.i.) (median = 120, range 80–180).

### Image analysis

Results of all on-site open readings and the associated data were returned on an Excel form to ANSM by all the centres. Any focal uptake higher than adjacent background that did not correspond to an organ or structure with physiologic retention of PSMA-11 was considered evocative for PC tissue (lesion). PSMA-11 PET/CT result was quoted positive, negative, or equivocal respectively if at least one evocative lesion, no lesion, or an equivocal lesion was found. Lesions were classified by regions which were prostate fossa including seminal vesicles, pelvic lymph nodes, distant lymph nodes, and other distant organs. For each positive examination, the reader reported the spread of the foci evocative of PC lesions in those regions. In case of foci evocative of metastases out of the prostate fossa, the reader quoted their number according to the scale: 1–3, 4–5, or 6 and over. In case of multiple image acquisitions, the reader also quoted the added value on the diagnostic thinking of early or delayed images compared with the interpretation based on images obtained between 50 and 100 min after injection, the “standard” time point.

### Statistics

The statistical analysis was performed using the software “MedCalc” (Ostend, Belgium) with a significance level of *p* < 0.05. The exact 95% confidence interval (CI) of rates was calculated. The qualitative variables were compared using the chi-square test, and the quantitative variables were compared using the Kruskal-Wallis test. Differences between the modalities of a variable were only search if the global test was significant. A multivariate stepwise logistic regression was used to assess the association between PET/CT results (positive vs. negative or equivocal) and variables whose results were available before PSMA-11 PET/CT. Receiver operating characteristic (ROC) curve analysis was carried out and the area under the curve (AUC) was determined to evaluate the predictive value of the multivariate criterion on PSMA-11 PET/CT positivity. Youden’s index was calculated to determine the cut-off value that gave the best combination of sensitivity and specificity for this prediction.

## Results

### Positivity rate of PSMA-11 PET/CT (**Tables**2 and 3)

The overall patient-based positivity rate (PR) was 742/1084 = 68% (CI 66–71%); 99/1084 = 9% (CI 7–11%) of PET/CTs were equivocal, and 243/1084 = 22% (CI 20–25%) were negative. The PR was identical if only the 1st PSMA-11 PET/CT was considered for the 32 patients who had two examinations: 720/1052 = 68% (CI 66–71%).

**Table 2:** Patient-based positivity rate of PSMA-11 PET/CT according to patients’ characteristics before PSMA-11 PET/CT

	Nb PSMA-11 PET/CTs	PSMA-11 negative	PSMA-11 equivocal	PSMA-11 positive
Overall	1084	22%	9%	68%
Gleason score (*n =* 1007)				*χ*^2^ *p = 0.13*
< 7	179	28%	8%	63%
= 7	718	22%	10%	68%
> 7	199	20%	6%	73%
Initial therapy				*χ*^2^ *p < 0.0001*
Prostatectomy (PX)	877	25%	10%	64%
Other curative modality	207	10%	5%	85%
Previous recurrence				*χ*^2^ *p = 0.01*
No (1st recurrence)	433	26%	10%	63%
Yes	651	20%	8%	72%
serum PSA for PSMA-11 PET/CT (ng/mL)				*χ*^2^ *p < 0.001*
< 0.2	17	41%	18%	41%
≥ 0.2 < 0.5	166	45%	13%	42%
≥ 0.5 < 1	173	27%	10%	63%
≥ 1 < 2	257	23%	11%	67%
≥ 2	471	12%	7%	81%
FCH PET/CT				*χ*^2^ *p < 0.001*
Negative	+924	24%	10%	66%
Equivocal	160	13%	5%	82%

**Table 3:** PSMA-11 PET/CT positivity rate according to the acquisition protocol

	Nb PET/CT examinations	Overall PSMA-11 positivity rate	PSMA-11 positivity rate for serum PSA < 0.5 ng/mL	PSMA-11 positivity rate for serum PSA < 1 ng/mL
Overall	1084	68%	42%	53%
PSMA-11 ponderal activity (MBq/kg)		*χ*^2^ *p = 0.5*	*χ*^2^ *p = 0.23*	*χ*^2^ *p = 0.21*
< 1.8	455	67%	34% (*n* = 67)	47% (*n* = 146)
1.8–2.2	477	69%	45% (*n* = 93)	55% (*n* = 163)
> 2.2	152	71%	52% (*n* = 23)	60% (*n* = 47)
CT contrast medium		*χ*^2^ *p = 0.7*	*χ*^2^ *p = 0.6*	*χ*^2^ *p = 0.7*
None	804	68%	41% (*n* = 123)	51% (*n* = 254)
Intravenous	206	71%	48% (*n* = 48)	57% (*n* = 77)
Oral	74	69%	33% (*n* = 12)	52% (*n* = 25)
Furosemide administration		*χ*^2^ *p = 0.56*	*χ*^2^ *p = 0.60*	*χ*^2^ *p = 0.15*
No	596	68%	40% (*n* = 110)	49% (*n* = 204)
Yes	488	69%	45% (*n* = 73)	57% (*n* = 152)
PSMA-11 PET/CT acquisition times		*χ*^2^ *p = 0.94*	*χ*^2^ *p = 0.90*	*χ*^2^ *p = 0.22*
One single at 50–100 min	254	67%	43% (*n* = 56)	51% (*n* = 98)
Post-injection and then 50–100 min	541	69%	40% (*n* = 94)	52% (*n* = 181)
Post-injection and then > 100 min	38	66%	NA (*n* = 5)	NA (*n* = 13)
50–100 min & then later	213	70%	NA (*n* = 22)	65% (*n* = 51)
Three time points	38	68%	NA (*n* = 6)	NA (*n* = 13)
Imaging protocol (if *n* ≥ 30)		*χ*^2^ *p = 0.14*	*χ*^2^ *p = 0.66*	*χ*^2^ *p = 0.17*
Acquisition post-injection and then 50–100 min, no contrast medium, no furosemide	460	69%	42% (*n* = 84)	56% (*n* = 157)
Acquisition at 50–100 min and then later, no contrast medium, with furosemide	174	74%	NA (*n* = 15)	70% (*n* = 37)
One single acquisition at 50–100 min, with IV contrast medium, with furosemide	131	69%	48% (*n* = 31)	55% (*n* = 51)
One single acquisition at 50–100 min, no contrast medium, with furosemide	76	63%	NA (*n* = 10)	47% (*n* = 30)
Acquisition post-injection and then 50–100 min, oral contrast medium, with furosemide	51	67%	NA (*n* = 9)	NA (*n* = 18)
Acquisition post-injection and then >100 min, no contrast medium, no furosemide	38	68%	NA (*n* = 5)	NA (*n* = 13)

*NA* not applicable; calculations and tests were only performed if *n* ≥ 30

### Influence of the patient’s characteristics on PR

Results are detailed in **Table** **2**.

In summary, a statistically significant difference in PR was found according to:

- the initial radical treatment: in absence of prostatectomy, the rate of foci in the prostate fossa was significantly greater (*p* < 0.001). No statistically significant difference was observed for the other regions;
- whether the current BCR was the 1st recurrence or the patient experienced previous recurrence(s): PR of 63% vs. 72%, *p* = 0.01;
- the age at PSMA-11 PET/CT: mean was 68.3 years for negative, 67.1 for equivocal, and 69.9 for positive scans, the difference being statistically significant only between positive vs. negative or equivocal results (*p* < 0.001); and
- the sPSA for PSMA-11 PET/CT: mean was 1.62 ng/mL for negative, 2.48 for equivocal, and 3.90 for positive scans (*p* < 0.001). The sPSA was < 0.2 ng/mL in 17 patients (mean 0.12, median 0.13, range 0.03–0.18) with an overall PR of 41% (CI 18–65%). Of them, 16 had undergone prostatectomy. This low sPSA was taken into consideration when the ATU was granted, except for 3 patients whose sPSA was fluctuating around 0.2 ng/mL and for 2 patients whose hormonal therapy was started before PSMA-11 PET/CT, leading to a drop in sPSA below 0.2 ng/mL. The sPSA was < 2 ng/mL in 60 patients who had not undergone prostatectomy (mean 1.15, median 1.09, range 0.11–1.98), with a PR of 75% (CI 64–86%), the foci being detected in the prostate fossa in 48% of cases but also as an oligometastatic spread (1–3 foci) in 23% of cases.

No statistically significant difference in PR was found according to:

- the patient’s body mass (*p* = 0.8); and
- the GS (*p* = 0.12).

### Influence of the imaging protocol on PR (**Table****3**)

No statistically significant difference in PR was found according to:

- the type of PET/CT machine used for image acquisition (p = 0.1);
- the injected activity (p = 0.4) and ponderal activity (p = 0.4);
- whether or not a contrast agent was administered (p = 0.7);
- whether or not furosemide was administered (*p* = 0.6);
- the CT dose-length product (*p* = 0.3); and
- the number of image acquisitions per PET/CT examination: 169/254 = 67% for 1 single acquisition, 546/792 = 69% for dual, and 27/38 = 71% for triple acquisitions (*p* = 0.7).

By combining the possible sequences of acquisition and the possible complementary administration of contrast media and/or furosemide, 21 different imaging protocols were used, without an overall significant difference in PR (*p* = 0.14). The two most frequent imaging protocols in use were rather different (**Table** **3**) but resulted in no significant difference in PR (*p* = 0.27). The “simplest” protocol, consisting in one single acquisition between 50 and 100 min without administration of contrast medium and furosemide, was used in only 18 PET/CTs, yielding a somewhat lower PR of 10/18 = 56% (but CI 31–78% is large).

**Table** **3** also shows the PR according to the imaging protocol (when performed at least in 30 examinations) in patients with sPSA < 0.5 ng/mL or 1 ng/mL; no statistically difference was found.

An added value on diagnostic thinking compared to the interpretation of the images acquired 50–100 min after injection was reported:

- in 31/579 (5%) early acquisitions, consisting in an increased confidence in the neoplastic character of later visible foci (*n* = 7), or in detecting evocative foci which were not visible later (*n* = 17) (**Fig.** **1**), or in a decreased confidence in the neoplastic character of later visible foci (*n* = 7); and
- in 136/251 (54%) late acquisitions, consisting in an increase confidence in the neoplastic character of already visible foci (*n* = 121), or in detecting evocative foci which were not previously visible (*n* = 7), or in a decreased confidence in the neoplastic character of previously detected foci (*n* = 8).

### Extent of foci in case of positive PSMA-11 PET/CT (**Table****4**)

Foci evocative of recurrence were present in the prostate fossa in 284 PET/CTs (26%), confined to this site in 181 cases (17%), and present in locoregional pelvic lymph nodes in 372 PET/CTs (34%), confined to this site in 146 cases (14%).

**Table 4:** Region-based positivity rate of PSMA-11 PET/CT and metastatic extent according to patients’ characteristics before PSMA-11 PET/CT

	Nb PET/CT examinations	PSMA-11 positive with pelvic foci only	PSMA-11 positive with distant foci	Foci in prostate fossa	Foci in pelvic lymph nodes	Foci in extra-pelvic lymph nodes	Foci in the skeleton	Foci in visceral organs	1–3 metastatic foci	4–5 metastatic foci	≥ 6 metastatic foci
Overall	1084	34%	35%	26%	34%	20%	15%	4%	31%	6%	15%
Gleason score (*n* = 1007)											
< 7	166	40%	23%	36%	23%	13%	9%	1%	20%	4%	11%
= 7	660	33%	35%	23%	36%	21%	17%	4%	33%	6%	15%
> 7	181	33%	40%	29%	35%	23%	18%	6%	36%	6%	15%
Initial therapy											
Prostatectomy (PX)	877	29%	35%	18%	34%	20%	15%	4%	33%	6%	13%
Other curative modality	207	53%	32%	58%	33%	20%	16%	4%	23%	4%	20%
Previous recurrence											
No (1st recurrence)	433	41%	21%	33%	30%	11%	10%	2%	27%	3%	11%
Yes	651	29%	43%	21%	37%	26%	19%	5%	34%	8%	17%
Serum PSA for PSMA-11 PET/CT (ng/mL)											
< 0.2	17	41%	0%	18%	24%	0%	0%	0%	24%	0%	0%
≥ 0.2 < 0.5	166	28%	14%	12%	25%	6%	8%	1%	30%	1%	2%
≥ 0.5 < 1	173	34%	29%	29%	31%	14%	16%	3%	37%	7%	5%
≥ 1 < 2	257	35%	32%	23%	33%	16%	18%	2%	34%	7%	10%
≥ 2	471	35%	46%	36%	41%	30%	20%	7%	30%	6%	27%
FCH PET/CT											
Negative	924	32%	34%	24%	33%	21%	15%	4%	30%	6%	14%
Equivocal	160	45%	37%	36%	43%	18%	18%	7%	38%	3%	19%

Foci evocative of distant metastases were present in 420 PET/CTs (36%), localised in extra-pelvic lymph nodes in 220 cases (20%), in the skeleton in 167 cases (15%), and in visceral organs in 44 cases (4%). The visceral organs harbouring foci evocative of metastases were the lungs in 22 cases, the peritoneum in 13 cases, the liver in 3 cases, the pleura in 2 cases, the anorectum in 2 cases, the testis in 2 cases, and in 1 case each, vas deferens, adrenal gland, muscle, presacral fat, or subcutaneous tissue.

The rate of oligometastatic spread was more frequent than that of polymetastatic spread: 31% for 1–3 evocative lesions or 37% for 1–5 evocative lesions vs. 15% for 6 lesions or more (*p* < 0.001). In this last case, extra-pelvic distant foci were present in 90% of cases vs. 56% in 1–3 oligometastatic foci and 67% in 4–5 metastatic foci (*p* < 0.001).

### Relation with the results of FCH PET/CT (**Tables****2** and **4**)

**Fig. 3:**
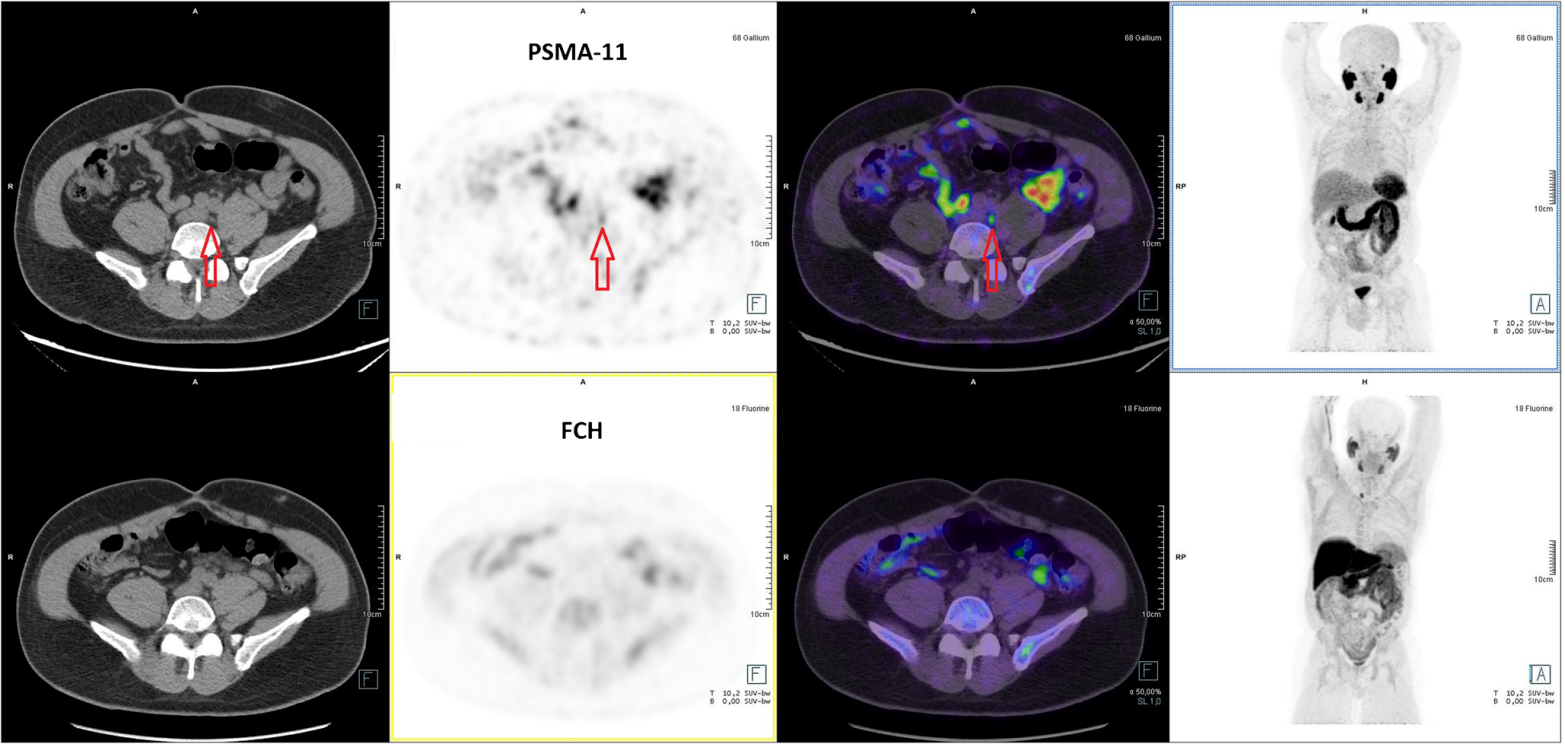
This 65-year-old patient underwent total prostatectomy. During follow-up, a rise in sPSA = 0.2 ng/mL prompted FCH PET/CT. **a** FCH PET/CT (lower row of images, from left to right: CT, PET, PET/CT fusion, MIP) was interpreted as negative for BCR. The FCH focus in the right lower part of the neck was interpreted as a probable hyperfunctioning parathyroid gland but no further exploration was performed. **b** PSMA-11 PET/CT (upper row of images) showed a significant 5-mm focus (arrow). Focused radiation therapy was performed on this lymph node and induced a drop in sPSA that persisted 1 year later, and the patient was accepted for a renal transplantation. This case illustrates the high sensitivity of PSMA-11 PET/CT to detect lymph node metastasis at sPSA as low as 0.2 ng/mL

ATUs were requested because a previous FCH PET/CT was negative in 924 cases, of which PSMA-11 PET/CT was also negative in 222 cases (24%), but positive in 611 cases (66%) (**Fig.** **3**) and equivocal in 91 cases (10%).

**Fig. 4:**
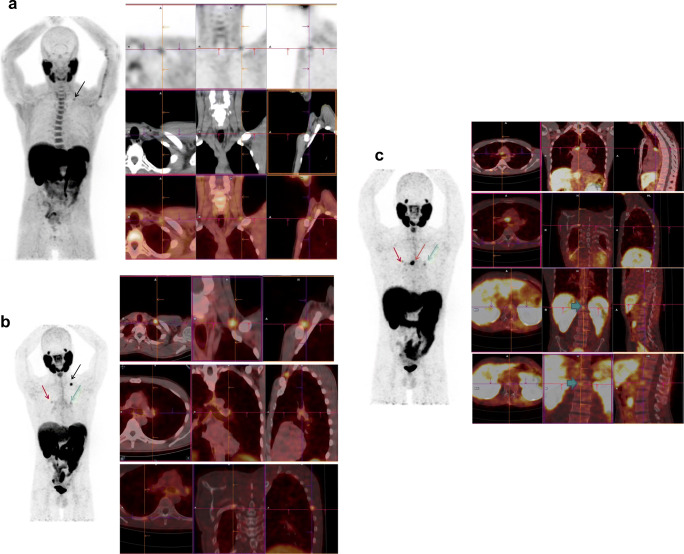
This patient with prostate cancer, Gleason 4 + 3, was treated by prostatectomy and radiotherapy. His first BCR after 2 years was treated by hormone therapy. His second BCR occurred 3 years later, sPSA = 1.2 ng/mL. **a** FCH PET/CT (from left to right: MIP, axial, coronal, and sagittal fused PET/CT slices) was then performed, showing a left focus of low intensity (SUVmax = 2.3) in a supraclavicular lymph node (black arrow), interpreted as equivocal. **b** On ^68^Ga-PSMA-11 PET/CT, a clear focus in the left supraclavicular lymph node (black arrow) was considered positive (SUVmax = 6.9), and 2 less intense foci were discovered in a left mediastinal lymph node (green arrow) (SUVmax = 2.6) and in the 7th right rib (red arrow) (SUVmax = 2.5), which were considered equivocal. Overall, the BCR was considered to be oligometastatic. Radiotherapy was performed on the left supraclavicular lymph node that induced a drop of sPSA. **c** Six months after the end of radiotherapy, sPSA increased at 4 ng/mL that prompted a follow-up PSMA-11 PET/CT. The irradiated left supraclavicular lymph node was no longer visible but the left mediastinal lymph node took up PSMA-11 more intensely (green arrow) and other mediastinal lymph nodes were now visible (SUVmax = 11.4) (brown arrow). The focus in the right rib was also more intense (SUVmax = 3.2) (red arrow), and two foci were discovered in the lumbar spine (bold arrows on coronal slices). The patient was then treated by hormone therapy. This case illustrates the high sensitivity of PSMA-11 to detect invasion of lymph nodes and the fact that any non-physiologic PSMA-11 focus should be taken into consideration, even with a moderate SUVmax

FCH PET/CT was equivocal in 160 cases, of which PSMA-11 PET/CT was also equivocal in 8 cases (5%), negative in 21 cases (13%), but positive in 131 cases (82%) (**Figs.** **1**, **2**, and **4**).

### Multivariate analysis

By performing a stepwise logistic regression, six predictors of a positive PSMA-11 PET/CT contributed significantly (all *p* < 0.001 except the GS *p* < 0.01) to the criterion *C*:

$$ \mathrm{LC}=\mathrm{logit}(C)=0.14624\ast \mathrm{sPSA}+0.76175\ast \mathrm{FCH}+0.029744\ast \mathrm{age}+1.2727\ast \mathrm{GS}-0.90596\ast \mathrm{initial}\ \mathrm{treatment}+0.50656\ast \mathrm{BCR}-1.8, $$

in which FCH is valued as 1 if FCH PET/CT was negative or 2 if equivocal, age is valued in years at PSMA-11 PET/CT, initial treatment is valued as 1 without prostatectomy or 2 if prostatectomy was undergone, and BCR as 1 for the 1st recurrence and 2 for previous recurrence(s). *C* value is (1 + exp(−LC))^−1^*.* The ROC analysis resulted in an area under the curve (AUC) of 0.70 (CI 0.68–0.73) (*p* < 0.0001 vs. 0.5). The threshold value of *C* = 0.67 yielded a positive predictive value (PPV) of 82% and a negative predictive value (NPV) of 48%. Another logistic regression including the same variables was performed, aiming to predict an oligometastatic pattern on PSMA-11 PET/CT, but AUC = 0.59, PPV = 74%, and NPV = 32% were low.

### Safety of PSMA-11

No adverse event was reported after the 1084 injections of PSMA-11, associated with 206 administrations of an intravenous contrast medium or 74 administrations of an oral contrast medium, in relation with the CT of PET/CT, and with 488 administrations of furosemide.

## Discussion

### Imaging protocol for PSMA-11 PET/CT

A procedure guideline for imaging with a ^68^Ga-labelled PSMA ligand was issued by EANM and SNMMI on March 2017 [16], 1 year after the first ATU was granted in France [14]. The recommended activity in the guideline is 1.8–2.2 MBq/kg of body mass. In our cohort, the mean and the median values (1.8 and 1.9 MBq/kg of body mass, respectively) fell into the lower half of this interval. However, there was no significant relation between the administered activity or ponderal activity and the PR (*p* = 0.6, **Table** **3**). This lack of relation between injected activity and PR was already reported in 2017 by Afshar-Oromieh et al.: median activities were 233 MBq (range 66–380) for positive PSMA-11 PET/CTs vs. 234 MBq (range 66–399) in case of negative scan [4].

In order to avoid activity in ureters that might lead to false-positive findings, the guideline proposes furosemide administration (20 mg i.v., shortly before or after administration of ^68^Ga-PSMA-11) after ruling out contraindications. This administration was performed in 488 PET/CT examinations of our series. Overall, the PR was not greater than without furosemide 69% vs. 68% (**Table** **3**). A positive non-significant trend was observed in 356 patients with sPSA < 1 ng/mL: PR was 57% with vs. 49% without furosemide (*p* = 0.15). However, in 183 patients with sPSA < 0.5 ng/mL, the difference was far from significance; PR was 45% with vs. 40% without furosemide (*p* = 0.60). Very few data are currently available on this topic. In 38 PET/CTs in patients with sPSA < 0.5 ng/mL, Haupt et al. [17] recently reported PR = 7/18 with furosemide, hydratation, and image acquisition 90 min p.i. vs. 5/20 without preparation and image acquisition 60 min p.i.

The administration of contrast media in 280 PET/CTs of our series did not result in a better PR, as its aim is a better landmarking of the lesions to organs and structures but not really to improve their detection.

According to the guideline, a 60-min interval is recommended for uptake time, with an acceptable range of 50 to 100 min. If this “standard” scan leads to indeterminate findings, the guideline advises performing a late scan at 3 h p.i. to identify lesions in close proximity to the ureter or the bladder, or lesions with a low PSMA expression and slower accumulation of the tracer [18]. The dominant practice in our cohort was somewhat different (**Table** **3**): one image acquisition was performed within the “standard” time interval of 50 to 100 min in 93% of the PET/CTs, but it remained as the single one in only 23% of the examinations. In the majority of cases (54%), early p.i. images were previously acquired for a better detection of spread in the pelvis (**Fig.** **1**). Kabasakal et al. observed, in 28 patients referred to PSMA-11 PET/CT for staging, that all the pelvic lesions detected at 60 min p.i. were better visible at 5 min p.i. [19]. Uprimny et al. [20] even found an added value to an early dynamic acquisition in 80 patients, 64 being referred for BCR. All the 55 lesions evocative of BCR on the delayed images at 60 min p.i. were already visible on the early images at 3 min p.i. without interference of the radioactivity in the urinary tract. Furthermore, the PR of local recurrence increased from 20% at 60 min p.i. to 30% with the early acquisition. In our series of 617 PET/CTs with an early p.i. acquisition, its added value, compared with the 50–100 min p.i. acquisition, appeared limited to 5% of the cases overall, mainly by showing foci evocative of malignancy which were not visible later (3%). An early acquisition should not be performed systematically, but might be indicated in case of equivocal FCH foci in the pelvis and/or in a clinical context leading to a high proportion of pelvic foci, i.e. according to **Table** **4**: first PC recurrence and/or a prostatectomised patient who did not receive complementary radiotherapy on the prostate bed (**Fig.** **1**).

A delayed acquisition in case of indeterminate findings was decided in only 22% of the PET/CTs, and its timing was clearly shorter than 3–4 h p.i. (median 2 h p.i.) (**Fig.** **2**). In contrast with an early acquisition, the decision of a complementary delayed acquisition may be taken in view of the results of the images at 50–100 min p.i. In 47% of the cases in whom it was performed, it added no information to the diagnostic thinking derived from the “standard” images. In the other cases, added information consisted mostly in confirming equivocal foci in 48% of the cases, whereas discovering previously undetected foci or a negative diagnostic thinking was limited to 5% overall.

Beheshti et al. [21] recently studied the influence of the timing of sequential image acquisition on the PR in BCR patients with sPSA < 1 ng/mL. The overall PRs in the same field-of-view were 28.4%, 39.8%, and 35.5% for dynamic (*n* = 135), 60 min p.i. (*n* = 135), and delayed (*n* = 97) phases, respectively. Additional images (i.e. dynamic, delayed, or both of them) provided more data for better delineation of the equivocal findings, resulting in positive final interpretation in 13.3% (18/135) or in negative final reading in 12.6% (17/135) of patients. The corresponding data of our series (**Table** **3** column sPSA < 1 ng/mL) do not correspond to systematic sequential acquisitions, since both early and delayed imaging was performed in only 26 cases, early imaging alone in 181 cases, and delayed imaging alone in 51 cases. Compared with the standard acquisition, the added information of those complementary acquisitions resulted in a positive result of PSMA-11 PET/CT in 38 cases (15%), mostly from delayed imaging in 31 cases, an equivocal result in 6 cases (2%), and a negative result in only 2 cases (1%). Although only the proportion of positive final interpretation is similar in the two studies, our results support the conclusion of Beheshti et al. [21]: “Overall, additional delayed scans were more helpful in better determination of the equivocal lesions.”

When integrating the timing of image acquisition with the optional administration of IV or oral contrast media or furosemide, 21 different imaging protocols have been used, according to the practice of the different PET centres, based on their experience of FCH PET/CT. No significant difference in PR was observed according to those imaging protocols (**Table** **3**), although the administration of furosemide and a dual-time acquisition at 50–100 min and later achieved a 70% PR in patients with sPSA < 1 ng/mL, a context which may favour this imaging protocol.

### Detection rate of PSMA-11 PET/CT in relation with sPSA and other predictors

The relation between PR and the sPSA at PSMA-11 PET/CT appears clearly in our study and in 6 large series of BCR (*n* > 200) published by different teams from several countries (gathered in order to avoid data resampling) whose patients were not selected on the results of FCH PET/CT [2, 4, 7–9, 13] (**Table** 5). The correlation coefficient between the median PSA level and the overall PR in those studies is 0.82 (*p* = 0.01). The overall PSMA-11 PR in a study is linked with its proportion of patients with high sPSA.

**Table 5:** Positivity rate of PSMA-11 PET/CT in large published series (*n* > 200) of patients from different centres with biochemical recurrence (BCR) of prostate cancer, according to serum PSA level assayed for PSMA-11 PET/CT

	Present study	Eiber et al. 2015 [2]	Afshar-Oromieh et al. 2017 [4]	Caroli et al. 2018 [13]	Ceci et al. 2019 [7]	Fendler et al. 2019 [8]	McCarthy et al. 2019 [9]
Origin	6 centres, France	Munich, Germany	Heidelberg, Germany	Meldola, Italy	Bologna, Italy	San Francisco and Los Angeles, USA	Perth, Australia
Number of PSMA-11 PET/CTs	1084	248	1007	314	332	635	238
Proportion of patients who underwent prostatectomy	80%	100%	82%	84%	98%	73%	64%
Main inclusion criterion (as complement to BCR)	Negative (85%) or equivocal FCH PET/CT. Delay < 100 days	Prostatectomy, no chemotherapy	Initial PET/CT of each patient at BCR	Negative (28%) or dubious FCH PET/CT	PSA between 0.2 and 2.0 ng/mL	No investigational therapy. PET/CT *n* = 443, PET/MRI *n* = 192	3 or fewer metastases on contrast abdominopelvic CT and bone scintigraphy
PSA median (range), ng/mL	1.7 (0.03–112)	1.99 (0.2–59.4)	2.2 (0.01–1237)	0.83 (0.003–80)	0.61 (0.2–2)	2.1 (0.1–1154)	2.55
Positivity rate							
Overall Pelvic confined/distant	68% (*n* = 1084) 34%/36%	90% (*n* = 248)	80% (*n* = 1007)	63% (*n* = 314) 37%/26%*	54% (*n* = 332) 25%/29%	75% (*n* = 635) 35%/40%	77% (*n* = 238)
PSA < 0.2 ng/mL Pelvic confined/distant	41% (*n* = 17) 41% / 0%	-	46% (*n* = 69)	27% (*n* = 33)	-	0% (*n* = 2**)	-
≥ 0.2 PSA < 0.5 ng/mL Pelvic confined/distant	42% (*n* = 166) 28%/15%	58% (*n* = 19)	46% (*n* = 108)	42% (*n* = 78)	38% (*n* = 138) 17%/21%	38% (*n* = 136)	51% (*n* = 63)
≥ 0.5 PSA < 1 ng/mL Pelvic confined/distant	64% (*n* = 173) 33%/31%	73% (*n* = 33)	73% (*n* = 119)	54% (*n* = 58)	54% (*n* = 92) 24% / 29%	57% (*n* = 79)	67% (*n* = 24)
≥ 1 PSA < 2 ng/mL Pelvic confined/distant	67% (*n* = 257) 35%/32%	93% (*n* = 72)	80% (*n* = 166)	75% (*n* = 68)	71% (*n* = 102) 27%/43%	84% (*n* = 89)	63% (*n* = 24)
PSA ≥2 ng/mL Pelvic confined/distant	81% (*n* = 471) 38%/51%	97% (*n* = 124)	92% (*n* = 509)*	95% (*n* = 77)	-	92% (*n* = 331)*	94% (*n* = 127)

*FCH* ^18^F-fluorocholine, *n* number of PET/CTs, *PSA* serum level of prostate-specific antigen

*Calculated from data in the article

**Excluded from the analysis by the authors

For sPSAs less than 1 ng/mL, our results of PRs were not significantly different with those of the 6 other studies. They were also very similar to those of the multicentre study of Calais et al. [5] (PR = 41% vs. 42% for sPSA = 0.2–0.5 ng/mL, *p* = 0.7; 60% vs. 63% for sPSA = 0.5–1 ng/mL, *p* = 0.6). These similar values for PR show that PSMA-11 PET/CT can detect BCR at low sPSAs, in patients selected on basis of a non-conclusive FCH PET/CT, without a reduction in diagnostic performance.

For sPSAs ranging 1–2 ng/mL, PR in our cohort was significantly lower than that of 3 of the 6 series [2, 4, 8]. For sPSAs ≥ 2 ng/mL, it was the lowest of all the 6 series. This may be explained by the patient selection in our study, resulting in 82% of previously negative FCH PET/CT. At those sPSAs, the gap of PR between FCH and PSMA-11 PET/CTs narrows. In the study of Cimitan et al., FCH PR was 43% for sPSA between 1 and 2 ng/mL and 81% for sPSA > 2 ng/mL [22]. Patients referred to PSMA-11 PET/CT after a non-conclusive FCH PET/CT and sPSA > 1 ng/mL are likely to correspond to cases with occult recurrence difficult to detect. Nevertheless, in our cohort, the greatest PR was still observed for sPSA ≥ 2 ng/mL. Overall, PSMA-11 PET/CT, based on a completely different uptake mechanism from enhanced metabolism of choline, brings added value in case of negative or equivocal FCH PET/CT, at any value of sPSA, including > 2 ng/mL.

A difference in PR between 149 cases of 1st BCR (PR = 46%) and 138 cases of BCR after salvage therapy (PR = 59%) was already reported by Ceci et al. [7]. By selecting, in our larger series, cases with sPSA < 2 ng/mL, as in the study of Ceci et al., at 1st BCR, the PR was lower (PR = 135/258 = 52% vs. PR = 224/355 = 63%, *p* = 0.01); the difference in PRs between the two studies was not significant. In both studies, local foci were significantly more frequent at 1st BCR.

We also observed a significant relation between PR and age at PET, which was searched for but not found in shorter series by two other teams [4, 13].

### Extent of foci in case of positive PSMA-11 PET/CTs

The characterisation of BCR as oligometastatic enables targeted therapies such as stereotactic ablative body radiotherapy (SABR) guided by PSMA-11 PET/CT [23].

In the study of McCarthy et al. [9], 238 BCR patients (median sPSA 2.55 ng/mL), who were previously restaged by conventional imaging, i.e. abdominopelvic CT and bone scintigraphy, underwent PSMA-11 PET/CT. By subtracting from the 132 cases with 1–3 PSMA-11 foci the 43 cases whose PSMA-11 foci were confined to the prostate bed or seminal vesicle(s), the rate of oligometastatic spread on PET/CT was (132–43)/238 = 37%, and the rate of polymetastatic spread 51/238 = 21%.

In the study of Roach et al. [6] (median PSA level 1.1 ng/mL, range 0.01–1.5), the foci were confined in the prostate fossa in 51/312 patients (16%), but were evocative of oligometastatic spread (1–3 foci) in 119 cases (38%) or of  polymetastatic spread (> 3 foci) in 60 cases (19%). In the study of Ceci et al. [7] (median sPSA 0.61 ng/mL, range 0.2–2), the detection of 1–3 oligometastatic foci was reported in 151/332 patients (46%), and of > 3 polymetastatic foci in 27/332 patients (8%). In our study, the rate of 1–3 oligometastatic foci was 32% and > 3 foci 21%. Overall, a close relationship can be observed in those studies between the ratio of oligo/polymetastatic rates (O/P) and the median sPSA. The median sPSA was 1.7 ng/mL in our cohort, and we found values for oligometastatic rate and O/P (0.60) similar to those of McCarthy al. [9] (O/P = 0.64) and Roach et al. [6] (O/P = 0.66), whereas by selecting BCR patients with sPSA < 2 ng/mL, Ceci et al. [7] more frequently observed an oligometastatic pattern (O/P = 0.85). We conclude that the discovery of oligometastatic spread on PSMA-11 PET/CT is more frequent at low sPSA.

Currently accepted criteria for BCR depend on the initial treatment. After radical prostatectomy, BCR is defined by a sPSA ≥ 0.2 ng/mL that rises on at least two consecutive measures at least 3 weeks apart. After initial external beam radiation therapy, the criterion is a rise ≥ 2.0 ng/mL above the nadir of sPSA that occurs more than 6 weeks after therapy completion [24, 25]. In our study, those criteria were not fulfilled at the time of PSMA-11 PET/CT by 81 patients. sPSA was < 0.2 ng/mL in 17 patients, of whom 16 had undergone prostatectomy. The PR in this group was 41%, concordant with the rate reported in some studies: 32/69 = 46% [4] or 14/35 = 40% [26] but somewhat greater than 27% (9/33 and 10/37) in two recent studies [13, 27] (*p* = 0.22). In our study, no distant foci were found in this group of patients, and the rate of pelvic lymph node foci was 24%, oligometastatic in all the cases (**Table** **4**). sPSA was < 2 ng/mL in 64 patients who had not undergone prostatectomy; PR in this group was as high as 75%. As expected, the PET foci were localised in the prostate fossa in almost half of them; nevertheless, PSMA-11 PET/CT showed an oligometastatic pattern in 23% of them.

In view of the concordance of our results with the recent literature, it seems that BCR may be suspected and may lead to PSMA-11 PET/CT at lower sPSAs than currently recommended [24, 25], to early detect limited recurrence of PC that may respond to locally targeted therapy.

### Relation with FCH PET/CT

As FCH was registered in France in 2010 and was rapidly widely used for PC imaging [28], the three published studies between 2014 and 2016 that demonstrated superior diagnostic performance of PSMA-11 over FCH to localise BCR formed the basis enabling ANSM to deliver ATUs [1, 10, 11].

The study protocol of Bluemel et al. [12] was similar to ours, but the series was 29-fold shorter. In 32 BCR patients with negative FCH PET/CT, a PR of 44% was reported, lower than 66% in our study (*p* < 0.01). In addition, 5 patients with equivocal FCH PET/CT underwent PSMA-11 PET/CT, positive in 3 cases, confirming the FCH suspicious foci in 2 cases. More recently, Caroli et al. [13] reported, in 88 BCR patients, selected on basis of an equivocal or a negative FCH PET/CT, a PR for PSMA-11 of 67%, similar to our result (69%, *p* = 0.8). As it could be expected, the PSMA-11 PR was significantly greater in cases with previous equivocal compared with negative FCH PET/CT, 82% vs. 66% (**Table** **2**).

### Multivariate analysis

We performed a logistic regression in search for a multivariate criterion derived from pre-PET data to predict a positive vs. a negative or equivocal result of PSMA-11 PET/CT. GS was selected among the predictors, with the lowest significance. Several authors who included GS testing did not find its significant relation with PR, but Calais et al. reported a greater PR in patients with GS > 7 [5] and Verburg et al. noted more frequent foci in pelvic lymph nodes in case of high GS [3]. Although a significant area of 0.7 in the ROC curve was obtained in our multivariate analysis, the NPV of 48% was not sufficient for advising against PSMA-11 PET/CT in case of low criterion value. Conversely, the PPV of 82% could reasonably favour PSMA-11 PET/CT in patients with criterion value > 0.67. Several teams recently elaborated nomograms, which do not incorporate the results of FCH PET/CT, in contrast with ours. Rauscher et al. [29] proposed two predictive models; a validation study [30] showed similar predictive values as ours, and the authors conclude that the prediction models should rather be understood as a tool for a positive decision towards a PSMA-ligand PET/CT examination. Another model integrating more clinical information has recently been proposed by Ceci et al. [31] achieving a PPV of 81%, similar to ours, but a greater NPV of 76%.

### Tolerance of PSMA-11

The lack of adverse effect following injection of ^68^Ga-PSMA-11 in our series was expected and is in accordance with the absence of adverse or clinically detectable pharmacological effects in any of the 1007 patients reported by Afshar-Oromieh et al. [4]. Accordingly, Fendler et al. [8] observed no grade 2 or higher adverse event associated with ^68^Ga-PSMA-11 administration in 635 patients; only grade 1 events were noted in 15 of 635 (2%) patients who were also administered furosemide and a contrast medium.

### Limitations of the study

The main endpoint of the present study is determination of PR, not sensitivity and specificity since a standard of truth (SOT) is difficult to obtain in case of recurrent PC; pathologic evidence is rarely obtained and a surrogate SOT based on the decrease of sPSAs in response to irradiation of the targets determined on imaging imposes a long-lasting follow-up which was not required by ANSM. PR as the endpoint was shared by several studies, including some recently published [3, 5, 7–9, 13, 17, 18, 31, 32] and is suited to our objectives. On per-patient basis in BCR, the difference between PR and the rate of true-positive results corresponds to the rate of false-positive results (FPR) since no true-negative result is expected. In the recent study of Fendler et al. [8], the patient-based PR was 475/635 = 75% and the patient-based FPR 17/217 = 8%, based on a composite standard of truth. Those important results illustrate the low value for FPR with PSMA-11 and the limited proportion of BCR patients in whom the determination of SOT is possible (34% in this study). Therefore, comparing the PR in a large series according to the clinical context and the imaging protocol seams a reasonable option to reflect actual differences in diagnostic performance.

Our analysis is based on the on-site open reading of PSMA-11 PET/CT. No masked reading was performed and we did not address the reproducibility of the reading. This is required for comparing the reproducibility of the interpretation of various imaging modalities in the same patients, which is not the context of our study. In the literature, the interobserver agreement was reported as almost perfect for PSMA-11 PET/CT [33–35], except for some visceral foci [33] in particular lung nodules [34], and was superior to that of conventional imaging [35].

Concerning the predictive factors for a positive PSMA-11 scan, we could not check the sPSA doubling time (DT) which was not recorded in the ANSM files; however, the predictive value of DT for a positive result of PSMA-11 PET/CT is controversial, reported by some authors [3, 7, 32], but unconfirmed by others [4, 8, 9, 13]. Whether patients recently received androgen deprivation therapy (ADT) was not recorded in our files; again, conflicting results have been reported about the influence of ADT on detecting BCR with PSMA-11 [4, 27, 36]. Since sPSA was not systematically assayed on the day of PSMA-11 PET/CT, sPSA value in the analysis has been obtained earlier in some patients, when the ATU was requested to ANSM. The actual sPSA on the day of PSMA-11 PET/CT may have thus been underestimated in some patients with a rapid progression of sPSA, leading to a potential overestimation of PR for low sPSA values.

## Conclusion

The analysis of this large prospective series confirms that PSMA-11 PET/CT is able to detect PC tissue in BCR patients, even in case of a recent negative or equivocal FCH PET/CT, at a large range of sPSA values. At low sPSAs < 1 ng/mL, the PR was similar to reported values in patients who did not undergo FCH PET/CT. Some PSMA-11 PET/CTs were performed in prostatectomised patients with sPSA < 0.2 ng/mL and detected oligometastatic BCR in 23% of cases. At higher sPSA, the PR of PSMA-11 increased but appeared somewhat lower than published for non-FCH selected cohorts, probably because FCH is more effective in detecting BCR in this context that resulted in selecting the most difficult cases for PSMA-11, still with a high PR. Some non-prostatectomised patients with sPSA < 2 ng/mL were imaged, resulting in a high PR partly due to frequent foci in the prostate fossa, as expected, but also to oligometastatic spread. These results, concordant with those of shorter series, are likely to favour a modification in the criteria accepted for BCR and to allow PET/CT imaging in case of rising sPSA before it reaches the currently accepted threshold, aiming to early detect and treat oligometastatic recurrences.

Other significant predictors for a high PSMA-11 PET/CT PR were found: high GS, no prostatectomy, previous BCR, higher age at PET, and FCH PET/CT result equivocal but not negative.

A pattern of 1 to 3 foci out of the prostate fossa evocative of an oligometastatic spread was frequently observed, in 31% of cases overall, fluctuating from 20 to 25% in patients with GS < 7 or PSA < 0.2 ng/mL or non-prostatectomised until 37% for sPSA between 0.5 and 1 ng/mL (**Table** **4**). It paves the way for targeted therapy.

Furthermore, this analysis found no significant difference in PSMA-11 PR according to the imaging protocol: injected activity adjusted to patient’s body mass, dose-length product, enhancement of CT with a contrast agent, administration of furosemide, or multiple time point acquisition. By determining the added value of complementary acquisitions to the “standard” 50–100 min p.i., we conclude that early acquisition should not be systematic but may be useful in patients with a high probability of BCR confined to the pelvis (prostatectomised patients without further local radiation therapy, generally at 1st BCR and/or equivocal FCH foci limited to the pelvis). A complementary delayed acquisition (on average at 120 min p.i.) was essentially useful for confirming equivocal foci on the “standard” acquisition.

It is not currently possible to define a “standard” imaging protocol that would be used systematically; it should rather be decided on an individual basis, among a limited number of alternatives.

## Data Availability

Yes, as an anonymous Excel table approved by the French Commission Nationale de l’Informatique et des Libertés (CNIL) on October 23, 2019, n° 2215516 v 0
